# Supplementary material for: Interaction of Graphene Oxide Modified with Linear and Branched PEG with Monocytes Isolated from Human Blood
Source: Nanomaterials (Basel). 2021 Dec 30;12(1):126. doi: 10.3390/nano12010126 (PMC8746718; doi:10.3390/nano12010126)
Supplement: Supplementary file 1 [file nanomaterials-12-00126-s001.zip › nanomaterials-1506561-supplementary.pdf]

nanomaterials

Supporting Information

Interaction of Graphene Oxide Modified with Linear and Branched PEG with Monocytes Isolated from Human Blood

Pavel Khramtsov [1],*, Maria Bochkova [1], Valeria Timganova [1], Anton Nechaev [2], Sofya Uzhviyuk [1], Kseniya Shardina [1], Irina Maslennikova [1], Mikhail Rayev [1] and Svetlana Zamorina [1]

[1] Institute of Ecology and Genetics of Microorganisms UB RAS, 13 Golev str., 614081 Perm, Russia; krasnykh-m@mail.ru (M.B.); timganovavp@gmail.com (V.T.); kochurova.sofja@yandex.ru (S.U.); shardinak@gmail.com (K.S.); i.maslennikova1974@gmail.com (I.M.); mraev@iegm.ru (M.R.); mantissa7@mail.ru (S.Z.)
[2] Institute of Technical Chemistry UB RAS, 3 Academician Korolev str., 614013 Perm, Russia; nechaev.a@itcras.ru
* Correspondence: khramtsovpavel@yandex.ru; Tel.: +7-342-280-77-94

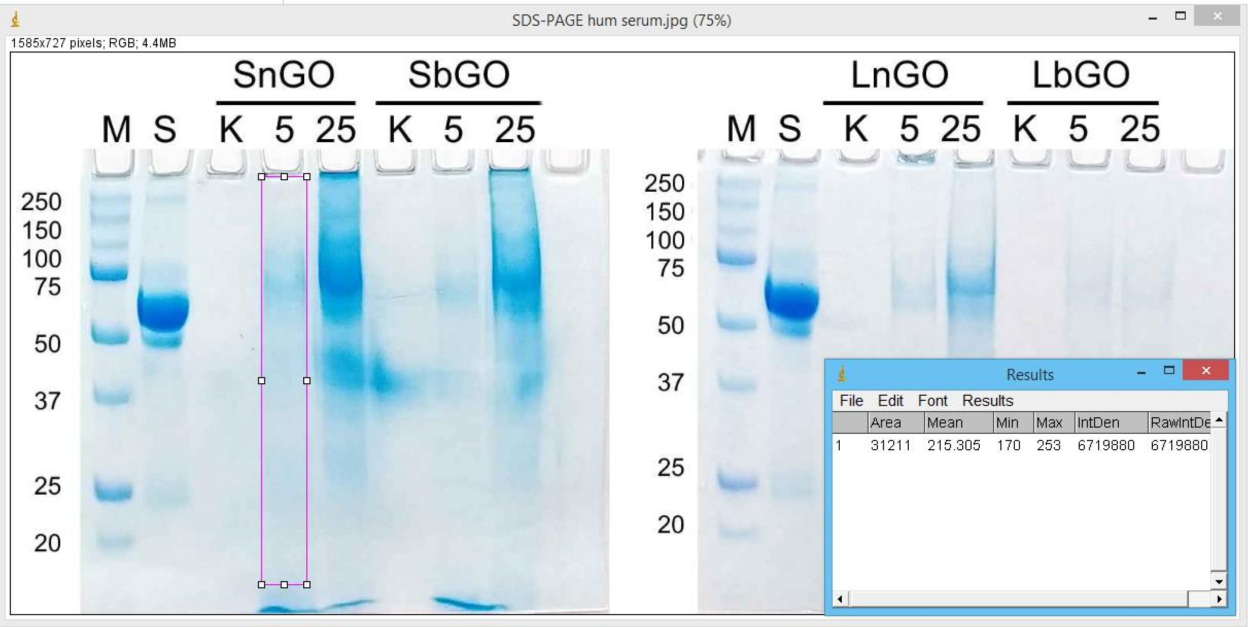

Figure S1. Measurement of the mean brightness of the SDS-PAGE lane using Image J. Pink frame indicates selected area. Mean grey value is displayed in the Results table.

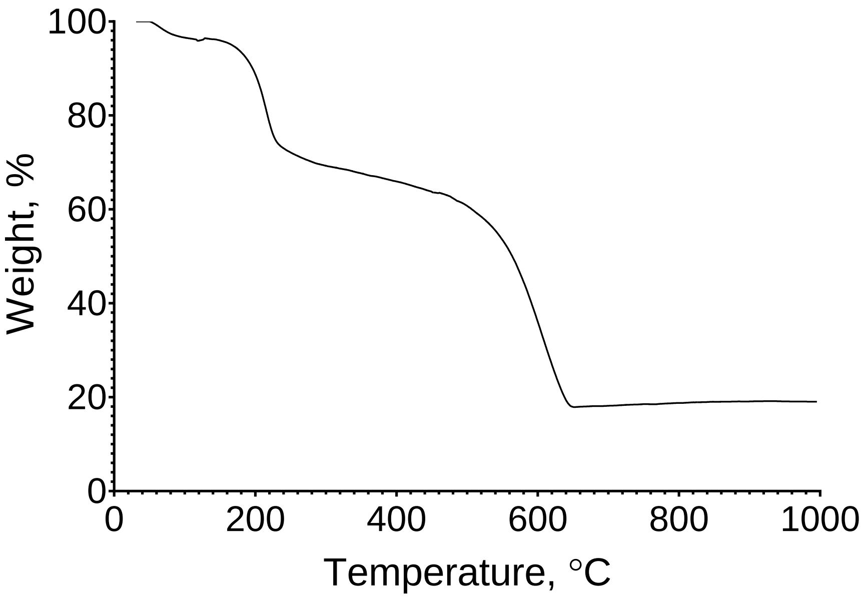

Figure S2. TGA curve of GO.

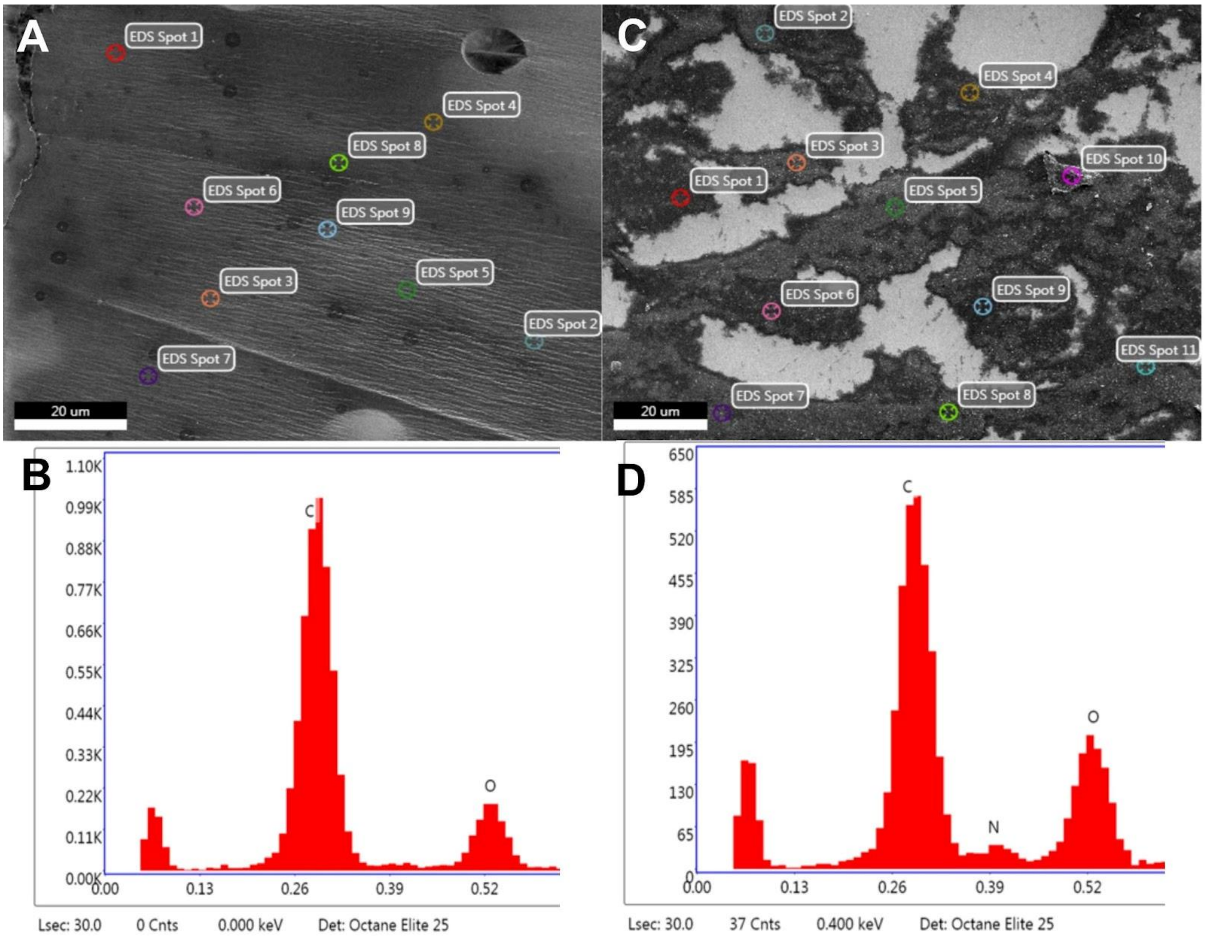

Figure S3. Mapping and EDS spectra of GO with lateral size of 100–200 nm (**A**,**B**) and SbGO (**C**,**D**).

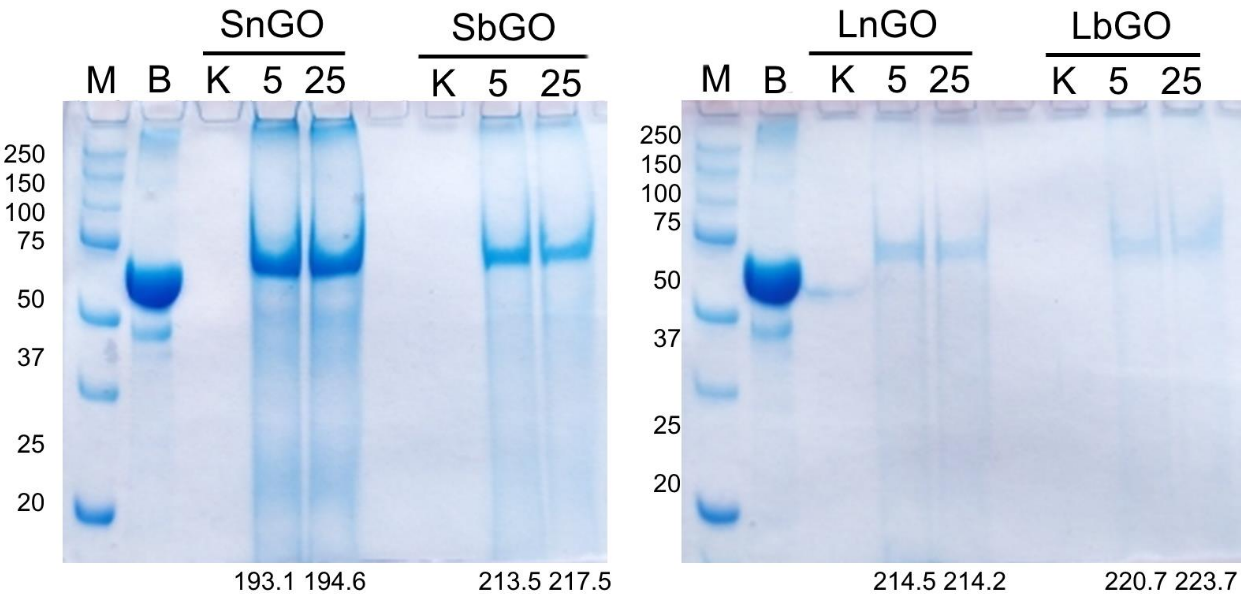

Figure S4. SDS-PAGE of GO-PEG incubated with BSA. M - protein markers, their molecular weights are indicated on the left side. B - 1% BSA. K - GO-PEG (25 µg/ml) incubated in culture medium without BSA. 5 and 25 - GO-PEG (5 or 25 µg/ml) incubated in culture medium with 7 mg/ml BSA. Mean gray values, which reflect amount of protein in sample, are presented below corresponding lanes.

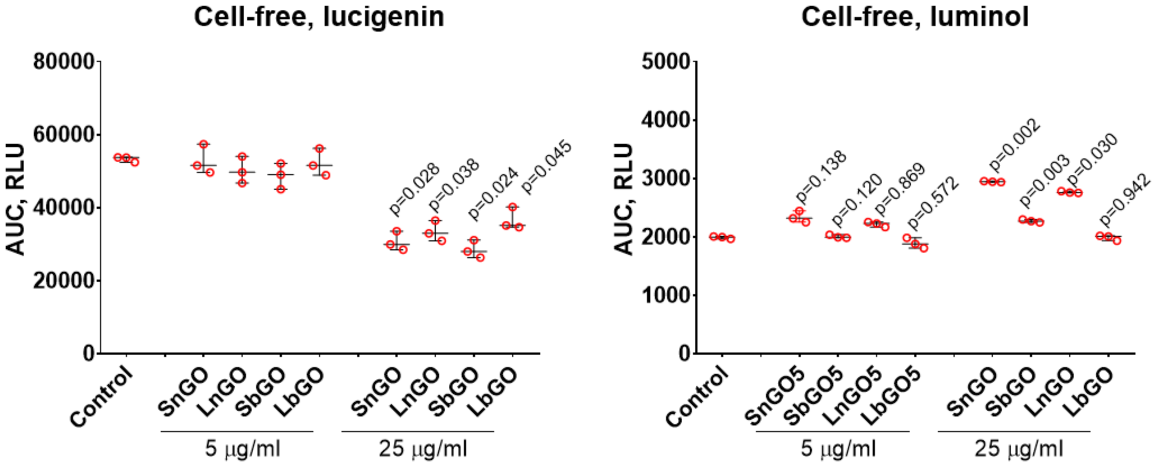

Figure S5. Luminescence of luminol (20 µM) and lucigenin (5 µM) induced by H_2O_2 (0.625% and 2.5%, respectively) in the presence of GO-PEG. Control - sample without GO-PEG. N = 3, median and interquartile range are reported. Data were analyzed by repeated measurements one-way ANOVA with Holm-Sidak post-hoc correction for multiple comparisons. *p* values indicate comparison to control sample.

Table S1. Concentration of endotoxin in GO-PEG, EU/mL.

	**5 µg/mL**	**25 µg/mL**
SnGO	2.3	11.5
SbGO	0.6	2.8
LnGO	0.3	1.4
LbGO	0.6	3.2
